# Cognitive Impact of Deep Brain Stimulation in Parkinson’s Disease Patients: A Systematic Review

**DOI:** 10.3389/fnhum.2022.867055

**Published:** 2022-05-13

**Authors:** Valentino Rački, Mario Hero, Gloria Rožmarić, Eliša Papić, Marina Raguž, Darko Chudy, Vladimira Vuletić

**Affiliations:** ^1^Department of Neurology, Faculty of Medicine, University of Rijeka, Rijeka, Croatia; ^2^Clinic of Neurology, Clinical Hospital Center Rijeka, Rijeka, Croatia; ^3^Faculty of Medicine, University of Rijeka, Rijeka, Croatia; ^4^Department of Neurosurgery, Clinical Hospital Dubrava, Zagreb, Croatia; ^5^School of Medicine, Catholic University of Croatia, Zagreb, Croatia; ^6^Department of Surgery, School of Medicine, University of Zagreb, Zagreb, Croatia

**Keywords:** deep brain stimulation, cognitive outcome, cognition, systematic review, Parkinson’s disease

## Abstract

**Introduction:**

Parkinson’s disease (PD) patients have a significantly higher risk of developing dementia in later disease stages, leading to severe impairments in quality of life and self-functioning. Questions remain on how deep brain stimulation (DBS) affects cognition, and whether we can individualize therapy and reduce the risk for adverse cognitive effects. Our aim in this systematic review is to assess the current knowledge in the field and determine if the findings could influence clinical practice.

**Methods:**

We have conducted a systematic review according to PRISMA guidelines through MEDLINE and Embase databases, with studies being selected for inclusion *via* a set inclusion and exclusion criteria.

**Results:**

Sixty-seven studies were included in this systematic review according to the selected criteria. This includes 6 meta-analyses, 18 randomized controlled trials, 17 controlled clinical trials, and 26 observational studies with no control arms. The total number of PD patients encompassed in the studies cited in this review is 3677, not including the meta-analyses.

**Conclusion:**

Cognitive function in PD patients can deteriorate, in most cases mildly, but still impactful to the quality of life. The strongest evidence is present for deterioration in verbal fluency, while inconclusive evidence is still present for executive function, memory, attention and processing speed. Global cognition does not appear to be significantly impacted by DBS, especially if cognitive screening is performed prior to the procedure, as lower baseline cognitive function is connected to poor outcomes. Further randomized controlled studies are required to increase the level of evidence, especially in the case of globus pallidus internus DBS, pedunculopontine nucleus DBS, and the ventral intermediate nucleus of thalamus DBS, and more long-term studies are required for all respective targets.

## Introduction

Parkinson’s disease (PD) is a widespread neurodegenerative disease that is most prevalent in individuals over the age of 65, posing a considerable burden on aging populations (Poewe et al., 2017). PD is a progressive disorder marked by motor symptoms like resting tremor, bradykinesia and rigidity, as well as non-motor symptoms such as sleep disorders autonomic dysfunction, behavioral changes and cognitive deficits (Vuletic et al., 2019). Additional research is elucidating the mechanisms underlying PD, which include the intracellular aggregation of α-synuclein, the formation of Lewy bodies (Bloem et al., 2021), and the loss of dopaminergic neurons. Damage of neurons starts in the olfactory bulb and locus caeruleus, but is most commonly followed in the substantia nigra dopaminergic neurons with further spreading throughout the brain as the disease progresses (Damier et al., 1999). This was postulated by Braak et al. (2003), who described a progressive escalation of pathology and symptom severity beginning in the lower brainstem and progressing to limbic and neocortical brain regions in the latter stages.

Research shows that PD patients have a significantly higher risk of developing dementia in later disease stages, leading to severe impairments in quality of life and self-functioning (Fang et al., 2020). Mild cognitive impairment is prevalent in PD patients, with a mean frequency of 27%, and many individuals develop to clinically severe dementia (Litvan et al., 2012). Additionally, the distribution of cognitive deficits in PD is centered on two distinct dopaminergic pathways in the frontal lobe and temporal lobes, with difficulties in planning, working memory, executive function, semantic verbal fluency and visual spatial ability (Fang et al., 2020).

The mainstay of treatment in early phases of the disease is focused on medicaments, although more invasive therapies may be employed in more advanced stages when medication alone cannot properly control symptoms (Armstrong and Okun, 2020). Deep brain stimulation (DBS) is a functional neurosurgical procedure that is used to treat movement, neurodegenerative and psychiatric disorders by modulating neuronal pathways (Lozano et al., 2019). It is typically used for treating motor symptoms in PD, while it is not as effective, or even aggravating, for gait, affective and cognitive symptoms (Mehanna et al., 2017). Common targets in PD include subthalamic nucleus (STN) and globus pallidus internus (GPi), while it is rarely used in ventral intermediate nucleus of thalamus (VIM) and pedunculopontine nucleus (PPN; Lozano et al., 2019). Current clinical practice of patient selection consists of patients with motor symptoms not controlled well with best medical therapy (BMT), while axial, speech, affective, and cognitive symptoms must be normal or minimally affected (Pollak, 2013). However, as the use of DBS broadens, a few questions remain. Mainly, what are the DBS effects on cognition, how to individualize therapy and reduce risk for adverse effects. The purpose of this systematic review is to review current knowledge in the field and to ascertain whether the findings have the potential to influence therapeutic practice.

## Methods

### Search Strategy

**FIGURE 1:**
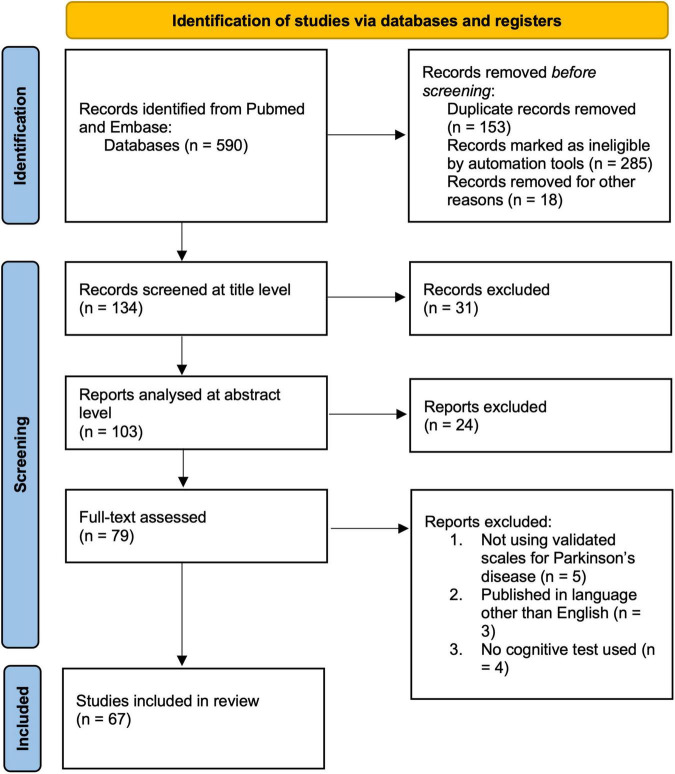
Prisma flow chart of the systematic review.

We have conducted a systematic review according to PRISMA guidelines (Page et al., 2021). Our search was focused on the MEDLINE and Embase databases. The search was done on articles published up to December of 2021. We used the following keywords on all fields and MeSH terms: “deep brain stimulation,” “Parkinson’s disease,” “cognitive effects,” “cognitive impact,” “cognitive outcome,” “cognition,” “subthalamic nucleus,” “globus pallidus internus,” “pedunculopontine nucleus,” “ventral intermediate nucleus of thalamus,” along with Boolean terms “AND” and “OR.” The search rendered 590 records after we applied appropriate filters. The studies were then selected based upon the following inclusion and exclusion criteria (Figure 1). Articles were first screened by title and abstract, followed by full-text checking for their eligibility. The selection of articles was done independently by four authors (VR, EP, MH, and GR), and final inclusion was done by agreement.

### Inclusion and Exclusion Criteria

Studies accepted for inclusion were: (a) studies with patients diagnosed with PD; (b) studies published up to December of 2021; (c) published in the English language; (d) published in indexed and peer-reviewed journals; and (e) evaluated cognition using validated scales and scoring systems.

Exclusion criteria include: (a) studies published in regional languages other than English, (b) no clear cognitive methodology or testing parameters described. Studies were checked for quality and finally, 62 studies were included (Figure 1).

## Results

The primary search yielded a total of 590 studies using the described method and search parameters. 134 studies remained after excluding duplicate records and filtering them out with automation tools. These were screened on the title level and 31 studies were excluded, leaving 103 studies that were analyzed on the abstract level, where additional 24 studies were excluded. The full text was analyzed for 79 studies, and additional 12 studies were excluded (not using validated scales for PD, *n* = 5; published in a language other than English, *n* = 3; and no cognitive test used, *n* = 4). Therefore, 67 studies were included in this systematic review according to the selected criteria (Supplementary Table 1). The complete PRISM flow chart for this systematic review is given in Figure 1. When looking at study designs, the search yielded 6 meta-analyses, 18 randomized controlled trials, 17 controlled clinical trials, and 26 observational studies with no control arms. The total number of PD patients encompassed in the studies cited in this review is 3,194, not including the meta-analyses.

### Cognitive Impact of Deep Brain Stimulation Treatment

#### Impact on Global Cognition

The impact of DBS on cognition can be viewed through changes in global cognitive functioning, as measured by scales such as the Mattis Dementia Rating Scale or the Mini-Mental State Examination, or specialized scales focusing on certain aspects of cognitive functioning. The reported studies on global functioning show somewhat opposing results. A meta-analysis by Combs et al. (2015) and Xie et al. (2016) revealed a statistically significant decrease in global cognition when comparing subthalamic nucleus DBS (STN-DBS) with BMT and globus pallidus internus DBS (GPi-DBS) patients, even though the overall change was not large. On the other hand, several randomized controlled clinical trials, controlled clinical trials, and observational studies found no changes in global cognitive functioning in their patients (Witt et al., 2004, 2008; Schüpbach et al., 2006; Smeding et al., 2006; Contarino et al., 2007; Klempírová et al., 2007; Heo et al., 2008; York et al., 2008; Okun et al., 2009; Weaver et al., 2009; Castelli et al., 2010; Daniels et al., 2010; Williams et al., 2011; Schuepbach et al., 2013; Asahi et al., 2014; Tang et al., 2015; Boel et al., 2016; Acera et al., 2019; Vats et al., 2019; You et al., 2020). When comparing targets, a meta-analysis by Wang et al. (2016) and a randomized controlled trial by Weaver et al. (2012) found that STN-DBS PD patients’ global cognition deteriorated more frequently than GPi-DBS patients, while several randomized controlled studies found no differences in global cognition or cognitive functional performance (Weaver et al., 2009; Odekerken et al., 2013, 2015, 2016). An observational study with the longest timeframe reported a decline in global functioning that was most pronounced up to the 9th year of treatment, and remained stable at the last tested period 14 years after surgery (Volonté et al., 2021). Researchers have also found that people who have lower global cognitive function scores before having STN-DBS are less likely to have good results after the procedure (Perriol et al., 2006; Tir et al., 2007; Tsai et al., 2009; Rinehardt et al., 2010; Witt et al., 2011; Fukaya et al., 2017; Acera et al., 2019).

#### Impact on Specific Cognitive Domains

Majority of the studies evaluated specific cognitive domains using specialized testing. The domains include language, executive function, attention, memory, and processing speed.

##### Language

Changes in language were the most reported in numerous studies. Looking at meta-analyses, statistically significant decline in semantic and phonemic fluency was found in STN-DBS patients compared to BMT (Xie et al., 2016; Wang et al., 2021) patients and GPi-DBS patients (Combs et al., 2015; Tan et al., 2016; Wang et al., 2016), as well as a part of the natural disease progression with no control arms (Parsons et al., 2006). The decline of verbal fluency was detected in several randomized controlled trials in the STN-DBS groups (Wojtecki et al., 2006; Witt et al., 2008; Okun et al., 2009; Weaver et al., 2009; Zahodne et al., 2009b; Daniels et al., 2010; Phillips et al., 2012; Ehlen et al., 2014; Pinto et al., 2014; Tramontana et al., 2015), although this was not seen in all studies that compared STN-DBS to BMT or GPi-DBS (York et al., 2008; Daniels et al., 2010; Odekerken et al., 2013, 2015, 2016; Rothlind et al., 2015; Boel et al., 2016). One study assessed the differences between STN-DBS and pedunculopontine nucleus DBS (PPN-DBS) patients and found that PPN-DBS patients experienced greater language deterioration in a small sample size (Pinto et al., 2014). Similar was seen in a study comparing localizations closer to VIM rather than STN, with increased verbal fluency deterioration (Ehlen et al., 2014). Slight declines of fluency in STN-DBS patients compared to BMT control patients was seen in several controlled clinical trials (Gironell et al., 2003; Smeding et al., 2006; Zangaglia et al., 2009; Castelli et al., 2010; Merola et al., 2011, 2014; Williams et al., 2011; Sáez-Zea et al., 2012; Foki et al., 2017; Szlufik et al., 2020; You et al., 2020), with only one study reporting no changes (York et al., 2008). The same trend was seen in observational studies as well (Funkiewiez et al., 2004; Castelli et al., 2006; Contarino et al., 2007; Heo et al., 2008; Schoenberg et al., 2008; Higginson et al., 2009; Fasano et al., 2010; Houvenaghel et al., 2015; Tang et al., 2015; Acera et al., 2019; Leimbach et al., 2019). Several studies that looked at language processing other than verbal or phonemic fluency found no significant changes in GPi-DBS (Rothlind et al., 2015) and STN-DBS patients (Heo et al., 2008; Castelli et al., 2010; Asahi et al., 2014; Rothlind et al., 2015; Foki et al., 2017).

##### Executive Functions

Executive function testing results published in the current meta-analysis reveal conflicting findings. Worsening of executive functions was seen in two studies, one comparing STN-DBS and GPi-DBS (Combs et al., 2015) and the other comparing STN-DBS to BMT control patients (Xie et al., 2016). On the other hand, two meta-analysis comparing STN-DBS and GPi-DBS (Tan et al., 2016; Wang et al., 2016), one that compared STN to BMT control patients (Wang et al., 2021), and lastly a single meta-analysis covering only STN-DBS patients found no significant alterations in executive function (Parsons et al., 2006). Four randomized clinical trials reported varying changes in executive functions, and interestingly, executive function was impaired in STN-DBS patients short-term (Daniels et al., 2010), but changes largely diminished in later time frames (Rothlind et al., 2015; Tramontana et al., 2015; Boel et al., 2016). Controlled clinical trials comparing STN-DBS to BMT control patients mostly reported no changes in executive function (Gironell et al., 2003; Smeding et al., 2006; York et al., 2008; Castelli et al., 2010; Foki et al., 2017), the exception was a two-part study with unilateral STN-DBS and GPi-DBS reporting decline in executive functioning compared to control patients (Zahodne et al., 2009a; Mikos et al., 2010). Similar findings were seen in a longer-term 3-year study comparing STN-DBS and BMT control patients (Zangaglia et al., 2009). Finally, non-consistent results can be seen in observational studies, with studies reporting either slight changes or no worsening in the clinical course of STN-DBS patients (Dujardin et al., 2001; Perozzo et al., 2001; Funkiewiez et al., 2004; Castelli et al., 2006; Klempírová et al., 2007; Ory-Magne et al., 2007; Fasano et al., 2010; Asahi et al., 2014; Rizzone et al., 2014; You et al., 2020).

##### Processing Speed

Meta-analyses reveal inconsistent outcomes in terms of processing speed. A prior meta-analysis and an observational study involving solely STN-DBS patients reported no significant differences in processing speed (Castelli et al., 2006; Parsons et al., 2006), while one study found that STN-DBS improves reaction times (Temel et al., 2006). Similar results were found in two recent meta-analyses and a controlled clinical trial comparing STN-DBS and BMT control patients (Williams et al., 2011; Xie et al., 2016; Wang et al., 2021). Slightly worse performance on testing has been found in STN-DBS patients compared to GPi-DBS patients in several studies (Combs et al., 2015; Tan et al., 2016; Wang et al., 2016). This was also seen in randomized controlled trials by Weaver et al. (2009) and Rothlind et al. (2015). A decline in processing speed was also found in unilateral STN-DBS and GPi-DBS patients (Zahodne et al., 2009a; Mikos et al., 2010), as well as PPN-DBS patients (Leimbach et al., 2019).

##### Attention

Results of cognitive testing in the domain of attention are not frequently reported in studies. One meta-analysis comparing STN-DBS to BMT and one study with only STN-DBS patients found no significant change in DBS patients (Parsons et al., 2006; Xie et al., 2016; Wang et al., 2021). Some slight changes were observed comparing STN-DBS to GPi-DBS, with worse results in the STN-DBS groups (Combs et al., 2015; Wang et al., 2016). The vast majority of controlled clinical trials found no changes in STN-DBS patients compared to BMT PD patients (Gironell et al., 2003; Zangaglia et al., 2009; Castelli et al., 2010; Sáez-Zea et al., 2012; Merola et al., 2014; Foki et al., 2017; You et al., 2020), with the exception of single study finding worsening of symptoms (Smeding et al., 2006). A randomized controlled trial by Tramontana et al. (2015) revealed worsening at the first control visit 12 months after the procedure, that largely diminished 24 months after the procedure, while Dafsari et al. (2020) found improvement in attention after STN-DBS compared to BMT PD patients.

##### Memory and Learning

The cognitive domain of memory was assessed in numerous studies, with conflicting results. Meta-analyses generally point to a slight decline in working and general memory mostly in STN-DBS patients compared to GPi-DBS and BMT patients (Combs et al., 2015; Wang et al., 2016; Xie et al., 2016), with only a study by Wang et al. (2021) showing no significant changes, but a trend of worsening in STN-DBS patients compared with BMT. More conflicting findings are found in randomized controlled trials and controlled clinical trials, with slightly fewer studies finding worsening of memory and intact learning (Smeding et al., 2006; York et al., 2008; Weaver et al., 2009, 2012; Rothlind et al., 2015), and no changes compared to BMT controls or between STN-DBS and GPi-DBS patients (Gironell et al., 2003; Zangaglia et al., 2009; Castelli et al., 2010; Daniels et al., 2010; Merola et al., 2011, 2014; Sáez-Zea et al., 2012; Tramontana et al., 2015; Boel et al., 2016; Foki et al., 2017).

##### Visuospatial Functions

Visuospatial function testing reveals no change in STN-DBS vs BMT patients in all meta-analyses (Xie et al., 2016; Wang et al., 2021), as well as most randomized clinical trials (Weaver et al., 2009; Tramontana et al., 2015), and controlled clinical trials (Gironell et al., 2003; York et al., 2008; Merola et al., 2011; Acera et al., 2019). One randomized controlled trial reported a slight decline in STN-DBS patients compared to GPi-DBS patients (Weaver et al., 2012), that was not seen in a different trial during a similar time frame (Boel et al., 2016). Three controlled clinical trials and one observational study reported improvement in visuospatial function and visuoconstructional task (Schoenberg et al., 2008; Zahodne et al., 2009a; Mikos et al., 2010; You et al., 2020).

### Impact of Deep Brain Stimulation Cognitive Change on Quality of Life

Quality of life is an important metric for measuring treatment benefits and the impact of possible adverse effects. DBS led to an increase in overall quality of life in both meta-analyses that reported results in this category, one of which reported a more significant improvement in GPi-DBS patients, and the other reported no changes between two targets (Tan et al., 2016; Wang et al., 2016). The same was found in two randomized controlled trials, one comparing STN-DBS and GPi-DBS, and another comparing STN-DBS with BMT patients (Smeding et al., 2006; Weaver et al., 2012). One randomized controlled trial focusing on early DBS intervention found significant improvement in quality of life in STN-DBS patients compared to BMT (Schuepbach et al., 2013). Quality of life appears to be connected to baseline cognitive functioning, as lower baseline functioning was related to worse outcomes in several studies (Zahodne et al., 2009b; Witt et al., 2011; Gruber et al., 2019). Furthermore, STN-DBS led to a reduction of quality of life when measuring communication, which was related to declines in fluency (Zahodne et al., 2009b). Importantly, this decline did not have a meaningful effect on daily activities in quality of life in the long term (Contarino et al., 2007).

## Discussion

Motor improvement post-DBS is well known and described in the literature (Bratsos et al., 2018). When looking at the effects of DBS on cognition, much is still uncertain due to some limitations in the field. It is clear that there is a profound difference in the number of patients when taking possible targets into account, with sparse studies of ventral intermediate nucleus of thalamus DBS (VIM-DBS) or PPN-DBS (Combs et al., 2015; Tan et al., 2016; Wang et al., 2016), which is a consequence of clinical practices and general preference for STN-DBS. Additional limitations in the field are a lack of long-term studies, as DBS can be used for more years by patients than present in the longest studies cited in this review (Hitti et al., 2019), and a small proportion of randomized controlled studies with large sample sizes. The primary questions addressed in this systematic review are how DBS affects cognition and whether this information can be utilized to guide individual therapy approaches, thereby avoiding potential detrimental effects.

With regard to global cognition, the studies with the strongest quality of evidence show that there is a slight decrease when comparing STN-DBS to BMT and GPi-DBS patients (Combs et al., 2015, 20; Xie et al., 2016). However, the majority of randomized controlled trials, controlled clinical trials and observational studies did not find any changes in global cognition, either when comparing STN-DBS and GPi-DBS, or STN-DBS to BMT, indicating that the overall effect is not large (Witt et al., 2004, 2008; Schüpbach et al., 2006; Smeding et al., 2006; Contarino et al., 2007; Klempírová et al., 2007; Heo et al., 2008; York et al., 2008; Okun et al., 2009; Weaver et al., 2009; Zahodne et al., 2009b; Castelli et al., 2010; Daniels et al., 2010; Williams et al., 2011; Asahi et al., 2014; Tang et al., 2015; Vats et al., 2019). This is further corroborated by studies that show no significant in the improvement of life quality, which is comparable in both targets (Smeding et al., 2006; Weaver et al., 2012; Tan et al., 2016; Wang et al., 2016). A long-term observational study covering patients up to 14 years after surgery reveled a decline in global functioning, however, the study was not controlled and overall declines in cognition are expected with aging (Volonté et al., 2021). Even so, it looks like both the STN DBS and the GPi DBS seem to be safe when it comes to cognitive function, with only small differences in performance that don’t have a big impact on quality of life.

Additionally, it is critical to emphasize the major findings on changes in specific cognitive functions. Most of the cited studies reported and assessed changes in fluency, with the vast majority of studies, including all meta-analyses, reporting a statistically significant decline in semantic or phonemic fluency in STN-DBS patients compared to both BMT and GPi-DBS patients (Gironell et al., 2003; Funkiewiez et al., 2004; Castelli et al., 2006, 2010; Smeding et al., 2006; Contarino et al., 2007; Heo et al., 2008; Schoenberg et al., 2008; Higginson et al., 2009; Zangaglia et al., 2009; Fasano et al., 2010; Merola et al., 2011, 2014; Williams et al., 2011; Sáez-Zea et al., 2012; Combs et al., 2015; Houvenaghel et al., 2015; Tang et al., 2015; Tan et al., 2016; Wang et al., 2016, 2021; Xie et al., 2016; Foki et al., 2017; Leimbach et al., 2019; Szlufik et al., 2020). Even greater deterioration of fluency has been described in VIM-DBS and PPN-DBS compared to STN-DBS, albeit in studies with a small sample, highlighting the need for further studies for these targets (Ehlen et al., 2014; Pinto et al., 2014). Importantly, there are indications that the deterioration in verbal fluency influences quality of life with regard to communication (Zahodne et al., 2009b), even though it did not have a meaningful effect on daily activities in a study by Contarino et al. (2007). These findings may have a significant impact on patient progression, and clinicians should be aware that DBS may result in fluency deficiencies when compared to BMT.

Findings for other specific cognitive functions are not as clear as fluency, with frequent contradictory findings. The deterioration reported in two meta-analyses that compared either STN-DBS and GPi-DBS, and STN-DBS to BMT patients, was slight, while five other meta-analyses did not show statistically significant changes in executive function (Parsons et al., 2006; Combs et al., 2015; Tan et al., 2016; Wang et al., 2016, 2021; Xie et al., 2016). Studies with longer-term follow-up had reported diminishing in executive function over time, even though this was also not consistent (Zangaglia et al., 2009; Rothlind et al., 2015; Tramontana et al., 2015). Comparing STN-DBS and BMT patients in processing speed and attention did not reveal significant changes (Williams et al., 2011; Xie et al., 2016; Wang et al., 2021), while slight deterioration is seen compared to GPi-DBS patients (Weaver et al., 2009; Combs et al., 2015; Rothlind et al., 2015; Tan et al., 2016; Wang et al., 2016). Similar is found in memory and learning, again a minor change in direct comparisons, but statistically significant for the majority of meta-analysis (Combs et al., 2015; Wang et al., 2016, 2021; Xie et al., 2016). On the other hand, randomized controlled trials and controlled clinical trials did have conflicting results, with the majority reporting no changes in memory (Gironell et al., 2003; Smeding et al., 2006; York et al., 2008; Weaver et al., 2009, 2012; Zangaglia et al., 2009; Castelli et al., 2010; Daniels et al., 2010; Merola et al., 2011, 2014; Sáez-Zea et al., 2012; Rothlind et al., 2015; Tramontana et al., 2015; Foki et al., 2017). Interestingly, studies that reported no changes had generally longer timeframes for endpoints, and the decline was more pronounced in STN-DBS rather than GPi-DBS. The effect of DBS on visuospatial functions seems to be more straightforward, as most of the studies revealed no changes in function when comparing STN-DBS and BMT patients (Gironell et al., 2003; York et al., 2008; Weaver et al., 2009; Merola et al., 2011; Tramontana et al., 2015; Xie et al., 2016; Wang et al., 2021), with slight differences in STN-DBS and GPi-DBS patients in one randomized controlled study (Weaver et al., 2012). Importantly, three studies highlighted improvements in visuospatial function and visuoconstructional task (Schoenberg et al., 2008; Zahodne et al., 2009a; Mikos et al., 2010). Taken together, these findings indicate that while the effects of DBS on specific cognitive functions are complex and variable, they almost always result in mild to moderate impairments in fluency, with conflicting findings for executive function, processing speed, attention, and memory, and no change or even slight improvement in visuospatial functions. The changes are less pronounced in GPi-DBS than in STN-DBS, PPN-DBS, or VIM-DBS, the latter two of which lack sufficient data to draw strong conclusions. Keeping this in mind can aid in patient selection for STN or GPi targets, even if the differences are minor.

Another key question is if we can lessen the likelihood of DBS having a detrimental effect on cognition. A randomized controlled trial by Witt et al. (2011) revealed that borderline global cognitive scores at baseline can lead to decreases in cognitive functioning after the procedure, significantly worsening the quality of life. The majority of patients repeatedly tested by the Repeatable Battery of Neuropsychological status remained stable, although patients who had difficulties in pre-surgical testing worsened after (Rinehardt et al., 2010). The importance of cognitive screening is also highlighted by several observational studies, as lower baseline global cognitive function was a predictor of worse outcomes in short and long term (Perriol et al., 2006; Tir et al., 2007; Tsai et al., 2009; Fukaya et al., 2017; Acera et al., 2019). All the studies mentioned in this paragraph are based on STN-DBS PD patients, and it would be interesting to see if GPi-DBS could be used to improve outcomes in patients who have slight cognitive impairment before the procedure.

It is relevant to highlight a potential limitation of our systematic review regarding the clinical applicability of the results, which is the potential relevance of genetic factors in cognitive outcomes of DBS therapy. Recent advances in the field of PD genetics highlight the potential importance of common risk and pathogenic variants such as GBA or LRRK2 on DBS outcomes, as unfavorable cognitive outcomes have been linked to GBA mutation carriers and certain LRRK2 phenotypes (Ligaard et al., 2019). This could allow for more personalized treatment based on a person’s genetics, and it should be looked into in both clinical trials and meta-analyses.

## Conclusion

This review shows that cognitive performance can decline in PD patients, and that even small changes can have an effect on daily quality of life. Current research is significantly skewed toward the effects of STN-DBS, followed by studies on GPi-DBS. The evidence thus far indicates that the highest occurrence of impairment can be seen in verbal fluency, while inconclusive evidence is still present for executive function, memory, attention, and processing speed. Global cognition does not appear to be significantly impacted by DBS, especially if cognitive screening is performed prior to the procedure, as lower baseline cognition is associated with worse outcomes. As a result, risk can be mitigated by tailoring the approach to each patient and testing cognitive function prior to the treatment. Additional randomized controlled studies are required to increase the level of evidence, especially in the case of GPi-DBS, PPN-DBS, and VIM-DBS, and more long-term studies are required for all respective targets.

## Data Availability Statement

The original contributions presented in the study are included in the article/Supplementary Material, further inquiries can be directed to the corresponding author.

## Author Contributions

VR, DC, and VV conceptualized the systematic review. VR, MH, GR, EP, MR, DC, and VV developed and consulted on the search strategy and methodology. VR, MH, GR, and EP assisted with screening articles. VR, MH, and GR abstracted data from the articles. VR drafted the manuscript. All authors reviewed, edited, assisted with writing subsequent drafts of the manuscript, and approved the final version of the manuscript.

## Conflict of Interest

The authors declare that the research was conducted in the absence of any commercial or financial relationships that could be construed as a potential conflict of interest.

## Publisher’s Note

All claims expressed in this article are solely those of the authors and do not necessarily represent those of their affiliated organizations, or those of the publisher, the editors and the reviewers. Any product that may be evaluated in this article, or claim that may be made by its manufacturer, is not guaranteed or endorsed by the publisher.
