# Supplementary material for: Cognitive Impact of Deep Brain Stimulation in Parkinson’s Disease Patients: A Systematic Review
Source: Front Hum Neurosci. 2022 May 13;16:867055. doi: 10.3389/fnhum.2022.867055 (PMC9135964; doi:10.3389/fnhum.2022.867055)
Supplement: Supplementary file 1 [file Table_1.pdf]

Supplementary Table 1. Cited studies listed by study design.

Author	Study design	Population	Follow-up	Main finding
Parsons et al. 2006	Meta-analysis	28 included studies covering only STN-DBS patients	Variable follow-up to 60 months	STN-DBS had small effects on general cognitive functioning, apart from verbal fluency (both semantic and phonetic). Attention, visual functions, processing speed and executive functions were not significantly affected.
Combs et al. 2015	Meta-Analysis	41 articles were reviewed with an aggregated sample size of 1622 patients (STN-DBS and GPi-DBS patients)	Variable follow-up (minimum 1 month, maximum 11 years)	STN-DBS – small declines in memory, attention, executive functions, processing speed, and overall cognition; and moderate declines were found in both semantic and phonemic fluency.<br><br>GPi-DBS - small declines in attention and small-moderate declines in verbal fluency.
Tan et al. 2016	Meta-Analysis	10 included trials with 1,034 patients (STN-DBS and GPi-DBS patients)	6, 12 and 24 months after surgery	GPi and STN DBS significantly improve advanced Parkinson's patients' symptoms, functionality, and quality of life. Statistically significant impairment on verbal fluency and processing speed in STN-DBS compared to GPi-DBS. No change in executive function, global cognition, language. Better quality of life was also obtained using GPi-DBS.

Wang et al. 2016	Meta-Analysis	Seven articles related to four randomized controlled trials that included 521 PD patients (STN-DBS and GPi-DBS patients)	36 months	Mattis dementia rating scale scores showed a statistically significant reduction in STN-DBS patients compared to GPi-DBS at thirty-six months post-DBS. STN-DBS patients performed slightly worse on average than patients in the GPi DBS group in attention, working memory and processing speed and phonemic fluency, with no significant differences in executive functioning and quality of life between the groups.
Wang et al. 2021	Meta-Analysis	Six articles that included 238 PD patients (STN-DBS and best medical therapy patients)	Variable (6- 36 months)	STN-DBS can lead to a moderate decrease of verbal fluency, in which the phonemic fluency declines greatly. The learning and memory function of the patients had a potential trend of weakening, but it was not statistically significant. Attention, concentration, executive functions, processing speed and visuospatial skills were not changed. The safety of STN-DBS is acceptable for most patients with motor symptoms.
Xie et al. 2016	Meta-Analysis	Ten articles including 797 PD patients (n=414 STN-DBS, n=383 best medical therapy)	Variable (6-24 months)	The results suggest that STN DBS results in slightly decreased global cognition, memory, verbal fluency, and executive function compared with control group. No significant difference is found in visuospatial function, attention and

				processing speed. STN DBS seems relatively safe with respect to cognitive function.
Schuepbach et al. 2013	Randomized controlled trial	251 PD patients, randomized to bilateral STN-DBS (n=124) and best medical therapy (n=127)	24 months	No significant change in global cognitive functioning. Improvement in overall quality-of-life.
Boel et al. 2016	Randomized controlled trial	124 PD patients, randomized to STN-DBS (n=62) and GPi-DBS (n=62)	24 months	No significant difference between STN-DBS and GPi-DBS in global cognitive functioning, executive function, verbal fluency and visuospatial function.
Odekerken et al. 2016	Randomized controlled trial	128 PD patients enrolled, 90 completed follow-up (47 GPi and 43 STN bilateral patients)	Three years	No difference in composite measures for cognition, mood and behavioral adverse effects at three-year follow-up
Okun et al. 2009	Randomized controlled trial	45 PD patients, randomized to bilateral STN (n=22) or GPi DBS (n=23)	7 months	No significant difference between STN and GPi DBS between pre-DBS to post-DBS seven-month cognitive performance. STN group exhibited greater decline on letter verbal fluency compared to the GPi group. No change in global cognition.
Philips et al. 2012	Randomized controlled trial	22 early PD patients (STN-DBS n=11, best medical therapy n=11) and 21 healthy control subjects	6 months	STN-DBS negatively impacted language in early PD, mostly grammatical and not lexical processing.

Odekerken et al. 2013	Randomized controlled trial	128 PD patients enrolled, (bilateral GPi-DBS n=65 and STN-DBS n=63)	12 months	No difference in composite measures for cognition, mood and behavioral adverse effects.
Rothlind et al. 2015	Randomized controlled trial	281 PD patients finished follow-up (best medical therapy n=117, bilateral STN-DBS n=84, GPi-DBS n=80)	6 months and 2 years	STN-DBS is associated with greater mean reductions in processing speed compared to GPi-DBS, while the latter being associated with lower performance on one measure of learning and memory.<br><br>The combined DBS group compared to best medical therapy has significantly greater mean reductions in performance on multiple measures of processing speed and working memory at 6- and 24-month intervals, while language, learning and executive functioning was not impaired.
Witt et al.2008	Randomized controlled trial	123 PD patients (best medical therapy n=63, bilateral STN-DBS n=60)	6 months	STN-DBS did not reduce overall cognition, learning or affectivity, although impairments can be seen semantic and phonemic verbal fluency, Stroop 2 naming colour error rate and Stroop 4 colour naming time.
Tramontana et al. 2015	Randomized controlled trial	30 early PD patients (best medical therapy n=15, bilateral STN-DBS n=15)	12- and 24-months	Modest reductions in attention, executive functions and word fluency at 12 months follow-up, that largely diminished at 24 months. No changes in visuospatial functions,

				working memory and naming.
Temel et al. 2006	Randomized controlled trial	39 PD patients (all bilateral STN-DBS	Concurrent testing	STN stimulation improves simple and complex reaction time in testing
Wojtecki et al. 2006	Randomized controlled trial	12 PD patients bilateral STN-DBS	Minimum 3 months post procedure	Low-frequency STN-DBS improves verbal fluency compared to high-frequency STN-DBS
Zahodne et al. 2009b	Randomized controlled trial	42 PD patients (n=20 unilateral STN-DBS, n=22 unilateral GPi-DBS)	6 months	Improvement in most subscales of quality of life except social support and communication. Changes in fluency contributed to reduced quality of life scores in communication in STN-DBS patients.
Weaver et al. 2009	Randomized controlled trial	255 PD patients (n=61 GPi-DBS, n=60 STN-DBS, N=134, best medical therapy)	6 months	The treatment was not associated with significant change on the Mattis dementia scale but other neurocognitive testing revealed small decrements in phonemic fluency, working memory and processing speed for DBS patients, while visuospatial function and learning was not affected.
Weaver et al. 2012	Randomized controlled trial	Total of 159 PD patients (STN-DBS n=70, GPi-DBS n=89)	3 years	Improved quality of life after procedure in both groups, no changes between. Slightly worse decline of global cognition in STN-DBS than in GPi-DBS. STN-DBS decline in visuospatial function, memory and learning.

Pinto et al. 2014	Randomized controlled trial	7 PD patients (all bilateral STN-DBS and PPN-DBS)	12 months	Stimulation of the PPNa, and possibly of surrounding structures as in the case of a spread of current to adjacent pathways, may contribute to the induction of stimulation-related speech deterioration.
Ehlen et al. 2014	Randomized controlled trial	38 subjects participated in the study, 13 with ET, 14 with PD and 12 healthy controls. All ET patients were treated by DBS in the vicinity of VIM, all PD patients with DBS in the vicinity of STN	2 months	Patients' verbal fluency performance was generally below normal. However, while activation of DBS in the vicinity of VIM provoked marked VF decline, it induced subtle phonemic VF enhancement in the vicinity of STN.
Daniels et al. 2010	Randomized controlled trial	123 PD patient (STN-DBS n=60, best medical therapy n=63)	6 months	Significant decline in STN-DBS only in executive function and verbal fluency 6 months after the procedure. Global cognition, learning, working memory and visuospatial functioning not affected. Patients with higher age, higher baseline LED, and/or higher axial subscore of the UPDRS at baseline have an increased risk for worsening.
Odekerken et al. 2015	Randomized controlled trial	128 PD patients enrolled (bilateral STN-DBS n=63 patients, bilateral GPi-DBS n=65 patients)	12 months	Class I evidence that there is no large difference in neuropsychological outcome between GPi DBS and STN DBS after 12 months.

Witt et al. 2011	Randomized controlled trial	119 PD patients (best medical therapy n=59, bilateral STN-DBS n=60)	6 months	Quality of life worsening could be related to lower cognitive functioning in patients with borderline global cognitive scores at baseline.
Dafsari et al. 2020	Controlled clinical trial	60 PD patients (STN-DBS n=40, GPi-DBS n=20)	Six months	Improved mood and cognition for both STN-DBS and GPi-DBS patients, more significantly in the latter. STN-DBS patients had improved attention and memory at follow-up.
You et al. 2020	Controlled clinical trial	40 PD patients (best medical therapy n=20, bilateral STN-DBS n=20) and 20 healthy controls	12 months	Significant decline in verbal fluency in STN-DBS compared to best medical therapy and controls, while visuospatial ability improved after STN-DBS. No significant differences in global cognition, attention and executive function.
Mikos et al. 2010<br><br>Zahodne et al. 2009a (same population)	Controlled clinical trial	43 PD patients (best medical therapy n=19, DBS groups of unilateral right STN n=3, left STN n=8, right GPi n=5, left GPi n=8)	16 months	DBS patients demonstrated greater individual decline on word list recall task, letter and animal fluency and several processing speed and executive function tests. Visuospatial functions improved in more patients.
Saez-Zea et al. 2012	Controlled clinical trial	21 PD patients (STN-DBS n=9, best medical therapy n=12)	6 months	Slightly worse deterioration in phonemic verbal fluency compared to controls, with attention and memory comparable.
Smeding et al. 2006	Controlled clinical trial	135 PD patients (bilateral STN-DBS n=99, best	6 months	STN-DBS group exhibited a larger decline in verbal fluency, color naming,

		medical therapy n=36)		selective attention and verbal memory compared to controls. No change in global cognition, executive functions, and an increase in quality of life.
York et al. 2008	Controlled clinical trial	51 PD patients (n=23 STN-DBS, n=28 best medical therapy)	6 months	Patients with STN-DBS exhibited significant decline in verbal memory and trends of decline in oral information processing at 6 months post-surgery. No changes in global cognition, learning, executive function, verbal fluency and visuospatial functions.
Rinehardt et al. 2010	Controlled clinical trial	40 PD patients (n=20 STN-DBS, n=20 best medical therapy)	4 months	Testing of the Repeatable Battery of Neuropsychological Status revealed that 94% of control patients and 73% of STN-DBS patients remained stable. Patients who had slight difficulties in pre-surgery testing worsened after.
Williams et al. 2011	Controlled clinical trial	37 PD patients (n=19 bilateral STN-DBS, n=18 best medical therapy)	24 months	No changes in global cognitive functioning. Patients with STN-DBS exhibited declines in nonverbal recall, oral information processing speed, lexical and semantic fluency. Number of patients who progressed to dementia did not differ from the control group.
Castelli et al. 2010	Controlled clinical trial	58 PD patients (n=27 bilateral STN-DBS, n=31	12 months	There was a significant decline in phonemic verbal fluency in the STN-DBS group, global cognitive functioning, executive

		best medical therapy)		functions, attention, memory and language was similar between the groups.
Merola et al. 2014	Controlled clinical trial	35 PD patients (STN DBS n=19, best medical therapy n=16)	Six years average follow-up	Similar progression of motor score and cognitive/behavioral alterations was observed between the two groups, apart from phonemic verbal fluency, which significantly worsened in STN-DBS patients. Memory and attention were unchanged.
Merola et al. 2011	Controlled clinical trial	40 PD patients, with either STN-DBS (n=20) or Duodopa enteral infusion (n=20)	15 months mean follow-up	STN-DBS was associated with a significant drop in the phonemic verbal fluency score. Memory and visuospatial functions were unchanged.
Zangaglia et al. 2009	Controlled clinical trial	65 PD patients (STN-DBS n=32, best medical therapy n=33)	Three years	Statistically significant worse verbal fluency and executive functioning in STN-DBS patients compared to controls, overall safe for general cognitive functioning, memory and attention.
Foki et al. 2017	Controlled clinical trial	43 PD patients (STN-DBS n=18, best medical therapy n=25, mild cognitive impairment patients n=24 and healthy controls N=12)	12 months	Roughly 10% of DBS patients showed cognitive decline in phonemic fluency compared to best medical therapy PD patients. Attention, language, memory and executive function unchanged.
Gironell et al. 2003	Controlled clinical trial	16 PD patients (unilateral pallidotomy n=8,	6 months	STN-DBS group had a clinically significant change in semantic verbal fluency, no changes in pallidotomy group. No

		bilateral STN-DBS n=8)		changes in memory, attention, visuospatial and executive functions.
Szlufik et al. 2020	Controlled clinical trial	Total of 54 patients, three groups of best medical therapy (20 patients), bilateral STN-DBS (20 patients) and late postoperative STN-DBS (14 patients)	30 months	Statistically significant deterioration in verbal fluency and working memory in the STN-DBS group, while learning and working memory deterioration was observed in the late-DBS group.
Acera et al. 2019	Observational study	50 PD patients (all bilateral STN-DBS)	5 years	No significant change in global cognitive functioning. Significant decline in verbal fluency and visuospatial ability during the tested period. Baseline functioning was the most significant predictor for cognitive decline.
Perriol et al. 2006	Observational study	58 PD patients (all bilateral STN-DBS)	12 months	20% of patients showed moderate worsening and 7% severe worsening in cognitive function on the Mattis Dementia Rating Scale, mostly those who had history of pre-operative cognitive and psychiatric comorbidities
Volonte et al.<br><br>2021	Observational study	18 PD STN-DBS patients (11 finished follow-up)	14 years	Global cognitive functioning worsened at 9 years compared to baseline but remained stable at 14 years post-surgery. Significant declines in semantic and phonemic fluency, no change in visuospatial functions.

Witt et al. 2004	Observational study	23 PD patients (all bilateral STN-DBS)	6- and 12-months	Short-term STN stimulation improved cognitive flexibility (giving up habitual responses) but impaired response inhibition. Long-term STN stimulation did not change global cognitive function.
Klempirova et al. 2007	Observational study	19 PD patients (all bilateral STN-DBS)	12 months	Global cognitive performance measured by Mattis Dementia Rating Scale was not significantly changed after STN-DBS. Logical memory and executive function worsened after the procedure.
Tang et al. 2015	Observational study	27 PD patients (all bilateral STN-DBS)	12 months	Diminished performance on a category fluency task, improvement in immediate recall task. No changes in global cognitive functioning.
Fasano et al. 2010	Observational study	32 PD patients (all bilateral STN-DBS, 20 finished the 8-year follow-up)	Up to 8 years	Slight declines in in verbal fluency, abstract reasoning, memory and executive function were found. Long-term cognitive morbidity of DBS was not significant.
Leimbach et al. 2019	Observational study	5 PD patients (all PPN-DBS)	12 months	No change in global cognition, memory and language. The only aspects of cognition that showed reliable decline in a proportion of the patients were some indices of processing speed (Stroop colour naming control task, WAIS-III digit symbol) and category switching verbal fluency.

Gruber et al. 2019	Observational study	104 PD patients treated with bilateral STN-DBS, long-term follow-up in 37 patients	Up to 10,5 years	Seven years post STN-DBS 41% of patients developed dementia or MCI with annual deterioration of 1.6/140/DBS-years via MDRS scale, while 18% remained normal functioning (comparable to normal disease course). Quality of life slightly declines post-surgery and correlated with diminished cognitive function.
Fukaya et al. 2017	Observational study	66 PD patients (all STN-DBS) split in two groups: independent functioning (n=33) and dependent functioning (n=33)	Five years average follow-up	General cognitive functioning diminished in both groups. Cognitive functioning at baseline was a predictive factor for outcomes.
Sheng-Tzung et al. 2009	Observational study	36 PD patients (all STN-DBS)	Mean follow-up of 31 months	Reduced cognitive function (MMSE<24) prior to DBS correlates with poor outcomes.<br><br>Patients with good cognitive function have prolonged positive outcome.
Contarino et al. 2007	Observational study	11 PD patients (all bilateral STN-DBS)	5 years	The decline of verbal fluency observed 5 years after implantation for DBS in STN did not have a clinically meaningful effect on daily living activities and quality of life. There was no global cognitive deterioration in the population with strict

				selection criteria for the procedure.
Schupbach et al. 2006	Observational study	29 PD patients (all bilateral STN-DBS)	18 to 24 months	No significant changes in global cognitive functioning as measured by the Mattis dementia rating scale. Patients had impaired social adjustment with difficulties in inter-personal relations.
Rizzone et al. 2014	Observational study	26 PD patients (all bilateral STN-DBS)	Up to 11 years	More than 70% had normal cognitive functioning at 11 years from surgery, while 22.7% of patients developed dementia. Highest number of patients worsened in executive functions.
Asahi et al. 2014	Observational study	11 PD patients (all bilateral STN-DBS)	12 months	DBS therapy did not significantly change global cognition, memory, visuospatial, executive functions and language, results of cognitive function were closely related to UPDRS part III.
Houvenaghel et al. 2015	Observational study	26 PD patients (all bilateral STN-DBS)	3 months	Slight postoperative impairment of phonemic or semantic fluency. The phonemic and semantic impairments appeared to result from the disturbance of distinct mechanisms.
Perozzo et al. 2001	Observational study	20 PD patients (all STN-DBS)	6 months	No significant changes in memory, attention and executive function due to STN-DBS.
Vats et al. 2019	Observational study	40 PD patients (all bilateral STN-DBS, young <65 yrs,	24 months	STN-DBS did not lead to any cognitive decline as

		elderly >65 years)		measured by the MMSE test regardless of age.
Castelli et al. 2006	Observational study	72 PD patients treated with bilateral STN-DBS	15 months	Statistically significant mild worsening in phonemic and semantic verbal fluency tasks in follow-up. No change in executive function and processing speed.
Ory-Magne et al. 2007	Observational study	45 PD patients (all STN-DBS)	24 months follow-up	No significant changes in general cognitive score, as well as fluency and executive functions in elderly patients.
Heo et al. 2008	Observational study	46 PD patients (all STN-DBS)	6- and 12-months follow-up	Minor decline in verbal memory and fluency, while there was no significant global cognitive and language.
Schoenberg et al. 2008	Observational study	20 PD patients (all STN-DBS)	5 months	Visuoconstructional task score improved, while semantic verbal fluency slightly declined. Global cognitive functioning, learning, visuospatial function and attention remained the same.
Dujardin et al. 2001	Observational study	9 PD patients (all bilateral STN-DBS)	12 months	At one year after surgery, most task measures did not change. Slight impairment was observed for tasks evaluating executive function. Examination of individual results showed that some patients (30% at 3 months after surgery) showed an overall cognitive decline.
Higginson et al. 2009	Observational study	22 PD patients (17 bilateral STN-DBS, 5	Average of 6.8 months	A proportion of patients experienced clinically significant worsening of

		staged placement)	after surgery	list learning and verbal fluency; majority of patients had no significant decline in cognitive performance.
Tir et al. 2007	Observational study	83 PD patients (all bilateral STN-DBS)	12 months	Cognitive decline was observed in 7.7% of patients, more significantly in patients who had lower baseline scores on the Mattis Scale (total n=14, decline in n=5, 36%)
Funkiewiez et al. 2004	Observational study	77 PD patients (all bilateral STN-DBS)	36 months	Decline in category fluency and total fluency was detected, with age being a predictor of decline in executive functions. There was no global cognitive decline.
